# Novel use of deucravacitinib for treatment of recalcitrant TIF1-gamma dermatomyositis

**DOI:** 10.1016/j.jdcr.2025.10.010

**Published:** 2025-10-14

**Authors:** Anuk Burli, Benjamin Gallo Marin, Elan Newman, Zunera Tahir, David F. Fiorentino

**Affiliations:** a Department of Dermatology, Stanford University, Stanford, California; b Sharp Rees-Stealy Medical Group, San Diego, California

**Keywords:** dermatomyositis, deucravacitinib, JAK inhibitor, TIF1-gamma

## Introduction

Dermatomyositis (DM) is a systemic autoimmune disease primarily affecting the skin and muscles, associated with dysregulation of type I interferon activity.^1^ Oral Janus kinase (JAK) 1-3 inhibitors such as tofacitinib and ruxolitinib have been associated with improvement of cutaneous disease in DM.^2^ However, JAK1-3 inhibition impacts a broad array of cellular signaling pathways and may precipitate various adverse effects, including infection, cardiovascular events, and thrombosis.^3^ Tyrosine kinase 2 (TYK2) is another member of the JAK signaling family that mediates signaling of a more limited number of immune receptors (including type I interferon) and thus may represent a more targeted option.^3^ We report here the use of a TYK2 inhibitor, deucravacitinib, for the treatment of a patient with refractory DM skin disease.

## Case

A 54-year-old woman with an 8-year history of DM with anti-transcription intermediary factor 1-gamma antibodies presented to our clinic with severe inflammatory skin disease. The patient was initially diagnosed with clinically amyopathic DM in 2016 but subsequently experienced muscle weakness and dysphagia, accompanied by mildly elevated creatinine kinase levels of 200s and magnetic resonance imaging consistent with myositis. Workup was negative for interstitial lung disease and internal malignancy. Although therapy with intravenous immunoglobulin (2 g/kg every 4 weeks), mycophenolic acid, and hydroxychloroquine improved her dysphagia and muscle weakness, she was unable to taper prednisone without a flare of her skin disease. Her skin inflammation was most notable on the scalp and bilateral temples, despite trials of medium-to-high-potency corticosteroids, calcipotriene 0.005%, crisaborole 2%, tacrolimus 0.1%, tapinarof 1%, ruxolitinib 1.5%, and compounded (2%) tofacitinib.

Prior treatments for the patient’s skin disease were associated with either lack of efficacy or adverse side effects. Methotrexate (20 grams orally weekly) and rituximab (1 g IV x 2 for 2 cycles) were of minimal benefit for her skin disease. She was unable to tolerate tofacitinib (5 mg orally twice a day) due to headaches and nausea.

**Fig 1:**
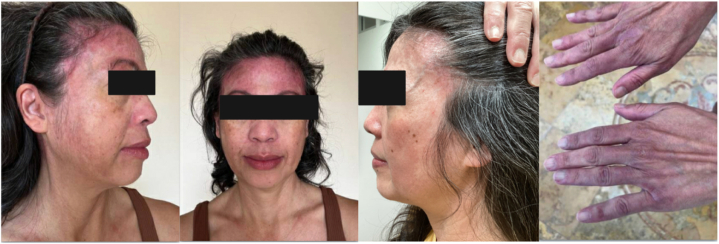
Dermatomyositis. Pre-deucravacitinib clinical photos.

Physical examination demonstrated well-defined erythematous, scaly plaques on the scalp and forehead and violaceous patches overlying the proximal and distal interphalangeal joints (Fig 1).

Photodistributed erythema of the chest and upper shoulders was seen along with facial erythema in a seborrheic pattern. She also presented with erythema of the eyelids and canthi associated with periorbital edema. Other significant findings in our patient included the presence of Gottron papules, lateral second digit hyperkeratosis, fingertip cracking, and periungual capillary dilation. Manual muscle testing demonstrated full strength in all groups except for mild neck weakness.

**Fig 2:**
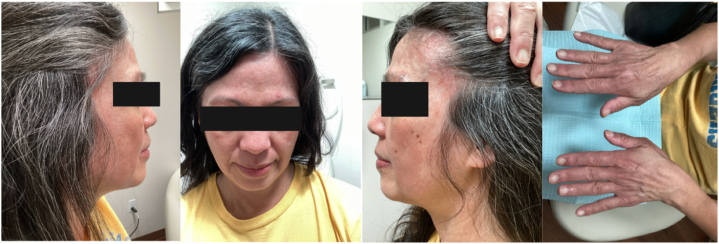
Dermatomyositis. Post-deucravacitinib clinical photos.

Given the inadequate control of her skin disease, she was started on deucravacitinib 6 mg daily and was maintained on intravenous immunoglobulin, prednisone 1 mg daily, and topical clobetasol and tofacitinib cream for the scalp and face, respectively. After only 1 month of therapy with deucravacitinib, she was able to discontinue mycophenolic acid without flare. At 5-month follow-up, the patient had continued improvement in her skin lesions in addition to significantly reduced pruritus and was able to taper off prednisone completely, along with sparing use of her as-needed topical medications (Fig 2). At 11 months of deucravacitinib therapy, her skin was almost completely clear with only a focal erythematous scaly plaque on the right posterior neck. The only adverse effect noted was mild acne on the forehead, controlled with topical salicylic acid 2% cleanser and clindamycin 1% gel.

## Discussion

This case reports significant improvement of DM skin disease associated with TYK2 inhibition (deucravacitinib). Interestingly, she did not experience the headaches or nausea associated with prior use of tofacitinib. Determining whether this drug may have been beneficial for myositis was not possible because the patient’s muscle inflammation was minimal prior to initiation of deucravacitinib. However, the patient reported less muscle pain while on the medication and did not have any clinical signs of myositis flares.

Deucravacitinib is currently approved by the Food and Drug Administration for moderate-to-severe plaque psoriasis, and, to our knowledge, its use in DM has not been reported. DM patients demonstrate dysregulation of the type 1 interferon signaling, which is mediated by TYK2 and JAK1.^1^ It is thought that the beneficial effects with the use of tofacitinib for DM skin disease may be related to its inhibition of interferon signaling (by interfering with JAK 1/3), although, since JAK1 mediates signaling by both type I and II interferons, the relative role for each is unclear at present. TYK2 does not mediate signaling by type II interferons, adding to the evidence that the type I interferon pathway might play a dominant role in DM pathogenesis. This is consistent with recent data demonstrating a dramatic response of DM skin disease associated with dazukibart, an anti-IFN-beta antibody that inhibits type I interferon signaling.^2^^,^^4^

Brepocitinib, a dual TYK2/JAK1 inhibitor, is being tested in a phase 3 clinical trial for DM (VALOR - NCT0543726).^5^ While not being evaluated for DM, deucravacitinib is currently being evaluated in a phase 3 clinical trial for cutaneous lupus erythematosus (POETYK SLE-2 - IM011-247).^6^

In clinical trials for psoriasis, the most common adverse effects associated with deucravacitinib were nasopharyngitis and upper respiratory infections, although more data are needed in larger patient populations to completely assess safety risk.^7^ Our case suggests that inhibition of TYK2 might represent a useful treatment strategy for patients with DM.

## Conflicts of interest

None disclosed.
